# A global approach to addressing the policy, research and social challenges of male reproductive health

**DOI:** 10.1093/hropen/hoab009

**Published:** 2021-03-21

**Authors:** Christopher L R Barratt, Christopher J De Jonge, Richard A Anderson, Michael L Eisenberg, Nicolás Garrido, Satu Rautakallio Hokkanen, Csilla Krausz, Sarah Kimmins, Moira K O’Bryan, Allan A Pacey, Frank Tüttelmann, Joris A Veltman

**Affiliations:** 1 Division of Systems Medicine, School of Medicine, Ninewells Hospital and Medical School, University of Dundee, Dundee, UK; 2 Department of Urology, University of Minnesota Medical Center, University of Minnesota, Minneapolis, MN, USA; 3 Section of Obstetrics and Gynaecology, MRC Centre for Reproductive Health, University of Edinburgh, Edinburgh, UK; 4 Department of Urology, Stanford University School of Medicine, Stanford, CA, USA; 5 IVI Foundation, Health Research Institute La Fe, Valencia, Spain; 6 Chair, Fertility Europe, Belgium; 7 Department of Biomedical, Experimental and Clinical Sciences “Mario Serio”, University of Florence, Florence, Italy; 8 Department of Pharmacology and Therapeutics, Faculty of Medicine, McGill University, and Department of Animal Science, Faculty of Agricultural and Environmental Sciences, McGill University, Montreal, Canada; 9 Department of Anatomy and Developmental Biology, School of BioSciences, Faculty of Science, University of Melbourne, Parkville, Australia; 10 Department of Oncology & Metabolism, University of Sheffield, Sheffield, UK; 11 Institute of Reproductive Genetics, University of Münster, Münster, Germany; 12 Biosciences Institute, Faculty of Medical Sciences, Newcastle University, Newcastle upon Tyne, UK

**Keywords:** male infertility, male contraception, male reproductive health initiative, research, policy, society

## Abstract

Male infertility is a global health issue; yet to a large extent, our knowledge of its causes, impact and consequence is largely unknown. Recent data indicate that infertile men have an increased risk of somatic disorders such as cancer and die younger compared to fertile men. Moreover, several studies point to a significant adverse effect on the health of the offspring. From the startling lack of progress in male contraception combined with the paucity of improvements in the diagnosis of male infertility, we conclude there is a crisis in male reproductive health. The Male Reproductive Health Initiative has been organized to directly address these issues (www.eshre.eu/Specialty-groups/Special-Interest-Groups/Andrology/MRHI). The Working Group will formulate an evidence-based strategic road map outlining the ways forward. This is an open consortium desiring to engage with all stakeholders and governments.

## Background and scientific rational

Male infertility is a global health problem. Meta-analysis data provide evidence for a decline in human testicular sperm production in many populations of around 1.6% per year between 1973 and 2011 (Levine *et al.*, 2017). Global statistics on the prevalence of male infertility indicate that up to 12% of men are infertile (Agarwal *et al.*, 2015, Cairo Consensus Workshop Group, 2020). Raising additional concerns are emerging reports showing that infertile men carry a higher disease burden, with an increased risk of incident disease (including heart disease and cancer) and die younger compared to fertile men (Kasman *et al.*, 2020a). Male infertility has therefore been termed a ‘harbinger of future morbidity and mortality’ (Stentz *et al.*, 2020) and is a health problem of growing global importance. Further, there is increasing evidence that the health of the father may have significant effects on the health of offspring (Kasman *et al.*, 2020b). Moreover, we now understand that the paternal epigenome can be significantly influenced by the environment and this can be manifested in the health of the offspring (e.g. Soubry, 2018). All of the aforementioned issues combine to provide a wake-up call for the crisis in male reproductive health and an urgent need to act (De Jonge and Barratt, 2019).

What is required to address these issues? Critical arguments for a strategic approach for Male Reproductive Health (MRH) have been presented by several authors (Barratt *et al.*, 2017, 2018, Skakkebaek *et al.*, 2019; De Jonge and Barratt, 2019; Ravitsky and Kimmins, 2019). An important starting point was to develop a global MRH Initiative (MRHI; De Jonge and Barratt, 2019) that aims to provide a road map to address emerging concerns for male reproductive health. To develop the MRHI road map, the authors have assembled as a multidisciplinary Working Group.

## Goals of the global male reproductive health road map

### What are the important time-sensitive research questions?

Remarkably, we still only have a basic understanding of the production, formation and function of a human spermatozoon. It is fundamental to understand these cellular, molecular, biochemical and genetic mechanism(s) to develop actionable intervention strategies and clinically relevant diagnostic assays that will assist in guiding more effective therapeutic approaches for male infertility.

There is evidence that poor male reproductive health is associated with poor overall health and early death (Kasman *et al.*, 2020a). What is the nature of this relationship? How can we untangle ‘cause and effect’ for this association?

How can we examine the impact of MRH on any offspring conceived naturally or through Medically Assisted Reproduction (MAR)? Moreover, compelling data are emerging that the environment (diet, toxicants and body mass index) can induce changes in the sperm epigenome and that these changes can be transmitted to the embryo to influence development and health (Donkin *et al.*, 2016; Ly *et al.*, 2017; Lismer *et al.*, 2020). This epigenetic contribution to the offspring suggests intergenerational biological effects driven by sperm. Important questions to address include (i) how does the epigenetic profile of the paternal genome become modified by environmental exposures such that health of the offspring is affected? (ii) is it possible to reverse environmentally induced epigenome changes to the sperm? (iii) does removal from environmental exposure(s) ameliorate the cause and effect or does the effect remain over multiple spermatogenic cycles or even a lifetime? and (iv) since it is well established that paternal aging contributes to genetic disease in the offspring, what are the mechanisms and are they treatable?

Work by other groups is contributing to formulating detailed key MRH research questions, for example, the WHO Expert Synthesis Group (Barratt *et al.*, 2017) and the Priority Setting Partnership for Infertility that has presented their top infertility research questions (Duffy *et al.*, 2020). In fact, it is essential that both broad and narrow scoping of literature data inform additional research questions to further support MRH.

### What experimental strategies can be used to answer the research questions?

While identifying research questions is a challenge, outlining realistic experimental design approaches to address them is significantly more difficult. Firstly, cohort investigational studies should be diverse in the ethnicity of men, geographic locations and socio-economic status. Further, how do we address some of the above questions in a real-world manner? How can current experimental methods be identified as effective? What limitations are present at experimental and organizational levels? What animal models might be helpful and in what context? How relevant are *in vitro* models and what new models of relevance could be developed? What existing data/repositories can address the questions (preclinical, genetic, national repositories, etc.)? What methodologies should be standardized and can investigational approaches be utilized from other disciplines? Is there an adequate resource of young investigators with appropriate training to address the growing research agenda? If not, how can a research career in MRH be promoted to reach the necessary capacity?

### Enhancing and suppressing male fertility

The burdens of both infertility treatment and fertility control continue to fall disproportionately upon women. New intervention strategies and treatment approaches aiming to improve male fertility would lead to a lesser requirement for women having to undergo MAR. What new treatments are in development and what barriers prevent progress?

The lack of effective, reversible male contraceptive methods is unacceptable, and it further perpetuates suppression of female freedom and economic growth in some regions of the world (Reynolds-Wright and Anderson, 2019). There is an urgent need and market demand for additional male contraceptive options but how can this be realized? There is a nascent resurgence of interest in male contraception under the auspices of, for example, the Bill and Melinda Gates Foundation (www.gatesfoundation.org/What-We-Do/Global-Development/Family-Planning) and NICHD (https://www.nichd.nih.gov/about/org/der/branches/crb). Moreover, there are now specific organizations for male contraception, for example, the Male Contraceptive Initiative (www.malecontraceptive.org/). Is there potential for synergy between the MRHI road map and these organizations?

### Global engagement of males, their partners and policymakers in MRH

Data show the need for greater involvement of men in their reproductive health and beginning early in their adult life (Hammarberg *et al.*, 2017, Prior *et al.*, 2019). For that to happen requires re-evaluation and, likely, redesign for how men are educated about their reproductive health. It also requires increased social awareness and education. This includes strategies to better engage men and their partners with, for example, medical and mental health professionals about MRH. Doing so will subsequently impact on public engagement, understanding and support. Collaborations with key stakeholders, such as Fertility Europe (http://www.fertilityeurope.eu/) and Resolve (https://resolve.org/), to examine information, research and strategy are necessary in this arena. Moreover, in this context, it would be valuable to assess the key strategies that have contributed to the success of campaigns aimed at examining male health in general, such as Movember (https://uk.movember.com) and Prostate Cancer UK https://prostatecanceruk.org/. What are the overlaps/synergies of MRH with wider issues in men’s health? There are a few established initiatives across the globe, for example, Global Action on Men’s Health (https://gamh.org/), Healthy Male (formerly Andrology Australia, https://www.healthymale.org.au/) and the Men’s Health Forum Ireland from the Republic of Ireland which became the first country in the world to publish a National Men’s Health Policy (https://www.mhfi.org/).

Affecting MRH research and education strategies is necessary but insufficient in isolation. Policy change is required. This involves forming fundamental strategic alliances, development of strategic methods and identification and assessment of funding streams (current and future). This necessitates discussion with a range of stakeholders, for example, policymakers, industry and funders. Establishing collaborations with government and health policymakers will drive associated funding agencies to invest in MRH. Moreover, we can learn by assessing how other disciplines have successfully addressed significant challenges to leverage complementary expertise and funding, for example, the Human Biomolecular Atlas (HuBMAP Consortium, 2019).

## Challenges to delivery of an MRHI road map and ways of mitigating their impact

### Resources

Any potentially socially transformative initiative is dependent on dedicated people, substantial time commitment and financial support. Informal discussions with key opinion leaders, professional societies (e.g. American Society of Andrology, Canadian Fertility and Andrology Society, IFFS, ASPIRE, Fertility Society of Australia, Society for Reproductive Biology (Australia and New Zealand)), funding agencies (e.g. NIH, Wellcome Trust) and industry strongly suggested that a global approach is needed and would attract significant support. However, almost all discussants wanted to first see the initiative develop and gain traction before they would consider committing resources or offer significant assistance. To address this, the MRHI submitted a grant proposal to the European Society for Human Reproduction and Embryology (ESHRE) for financial support to lay necessary groundwork for the initiative. The proposal was approved by ESHRE and the funds will be used by the Working Group to hold essential in-person meetings to galvanize the road map. Furthermore, the European Academy of Andrology (EAA) will provide support to assist with Working Group meetings and stakeholder conferences. Productivity outputs resulting from these funding sources will provide supportive evidence to better leverage more substantive and long-term funding from other sources, such as societies, funders, industry and private organizations. Development of a web presence is critical for visibility to promote the initiative, stimulate interest and create opportunities for collaboration (www.eshre.eu/Specialty-groups/Special-Interest-Groups/Andrology/MRHI). The goals are ultimately to globally influence society, education and legislative policymakers about the significance of MRH.

### Low starting base

Fundamentally, there is much intelligence gathering required. As illustrated by the WHO Expert Scanning Group analysis (Barratt *et al.*, 2017), the starting base is relatively low. Foundational data are required to build on our knowledge and even challenge whether our perceptions are correct. For example, is it correct to assume that a lack of funding for studies into the biology underlying male infertility (Table I) has negatively impacted upon diagnostic investigations and clinical management? In fact, what is the worldwide funding level for MRH? What clinical trials are ongoing? How effective is patient information on male infertility in different countries? What clinical cohorts and tissues repositories are available? Initial data addressing some of these questions are instructive (Table I; https://www.nihr.ac.uk/documents/reproductive-health-and-childbirth-specialty-profile/12117?diaryentryid=45412#Track_record). For example, examination of data from clinicaltrials.gov shows that there are 239 trials currently recruiting for female infertility and 223 trials currently recruiting for male infertility. However, of these ‘male infertility’ trials, 154 (69%) deal with female infertility, 20 (9%) deal with IVF and two deal with offspring only. Thus, there are 47 trials all over the world actively recruiting for male infertility compared to hundreds for female infertility.

**Table I:** Government funding for Male Reproductive Health (MRH^a^) research compared to total research funding, also expressed as a percentage of total funding and as funding per capita.

Country (agency and period)	Total funding for male reproductive health and per capita (US$)	Total funding commitment^f^ and per capita (US$)
Australia^c^ (NHMRC 2016)	$1.9 million (0.33%)	$578.5 million
	$0.08 per capita	$24.7 per capita
USA^d^ (NICHD 2019)	$39.5 million (0.26%)	$1.5 billion
	$0.12 per capita	$4.57 per capita
UK^b^ (MRC 2014–2016)	$1.7 million (0.17%)	$981.2 million
	$0.03 per capita	$14.95 per capita
Germany^e^ (DFG 2019)	$2.6 million (0.32%)	$812.3 million
	$0.03 per capita	$9.8 per capita

^a^ MRH in this context is confined to infertility and male reproduction and excludes, for example, cancers of the reproductive system. Data are from proximate funding periods and exclude industrial/commercial funding.

^b^ UK data are from the MRC (mrc.ukri.org). Four grants and one programme grant based on the classification ‘male fertility’ were awarded for research into: male infertility, semen analysis, sperm maturation and sperm motility. The Population and Systems Medicine Board (PSMB) spent in total £104 million on research in reproduction over the 3-year period. This constituted 3.6% of funding of PSMB for MRH compared to 9% for female reproductive health (with particular thanks to Dr Anabel Raszpla, MRC).

^c^ Australia data are from the NHMRC (www.nhmrc.gov.au). Compared to all NHMRC expenditure for 2016, Men’s Health (minus prostate) received 1.1% of the total NHMRC funding commitment for 2016. Encompassing the terms ‘fertile, fertility, infertile and infertility’, three grants were awarded in 2016 that related to Men’s Health. This equated to 0.4% of the overall 2016 funding commitment (with particular thanks to Niki Baxter NHMRC).

^d^ USA data are from the NICHD (www.nichd.nih.gov) for 2019. In 2019, $39,472,049 of funding was awarded for male reproductive health versus $111,922,746 which was awarded for female reproductive health. This is a similar funding ratio to that in UK at 1:3.

^e^ Germany (DFG 2019) data are from www.DFG.de (with particular thanks to Frank Tüttelmann).

^f^ Total Funding Commitment reflects total funding from MRC (UK), NHMRC (Australia), NIH (USA) or DFG (Germany) for comparable period. The sources of data are the websites of the respective granting bodies.

### Paucity of regional and national strategies

Given the limited activity in the MRH space to date, it is perhaps not surprising that there are only a few examples of a nationally or globally coordinated approach among the 193 UN Nation Member States. The Australian ‘Healthy Male’ (https://www.healthymale.org.au/), operating since 2000, was recently approached by the Australian Government to oversee the updating of the National Male Health Policy. This successful engagement reflected Healthy Male’s standing as a source of evidence-based quality advice. It is evident that MRH is a way of garnering attention in the health sector, which then allows a broadening of influence into other related health domains, especially regarding chronic disease and those underlying the social determinants of health, such as education, justice and employment

## Summary and conclusion

Male infertility is a spectrum of diseases of global significance. It, like many other conditions, will be impacted by genetic and environmental change; the identity of these insults and consequences, however, remain largely unexplored. In contrast to most other diseases, MRH has the potential to affect offspring health and, as such, the health of global populations. This, and the absence of safe and reversible male contraceptives, makes an investment in research and education an urgent priority of relevance to all genders. The current status quo leads to the burden of family planning and infertility treatment being unacceptably placed on the female partner. To address this gap, the authors have formed the MRHI. This is an open consortium desiring to engage with all stakeholders and governments.
